# Characteristic of HIV/AIDS infected patients at the detection and HAART initiation

**DOI:** 10.1186/1471-2334-14-S4-P14

**Published:** 2014-05-29

**Authors:** Tiberiu Holban, Ina Bîstrițchi, Constantin Spânu, Angela Nagîț

**Affiliations:** 1 Nicolae Testemițanu State Medical and Pharmaceutical University, Chişinău, Republic of Moldova; 2 National Public Health Center, Republic of Moldova; 3 Hospital of Dermatology and Communicable Diseases, Chişinău, Republic of Moldova

## 

We evaluated the clinical features, immunological and virological indices in HIV/AIDS–infected patients at the detection and antiretroviral therapy (HAART) initiation.

We followed up 149 adult patients diagnosed with HIV/AIDS infection between the years 1997-2011 and supervised in the specialized department of the Clinical Hospital of Infectious Diseases “Toma Ciorbă”. These patients received HAART since 2011. The late diagnosis is defined by the presence of AIDS associated diseases and/or the level of CD4 <350 cells/µL.

Studying general features of HIV infection depending on specific laboratory indices of this disease, was found that nearly two-thirds (63.09%) of patients with HIV/AIDS are detected late and more than half of them (59, 57%) have already advanced HIV infection which develop specific clinical manifestations of HIV/AIDS such as oropharyngeal candidiasis, tuberculosis and wasting syndrome, which represents a strong correlation between disorders of the immune system activity expressed by the considerable decrease in CD4 levels and facilitation to develop the opportunistic infections. The predominant route of HIV transmission was heterosexual in 87.25% of cases, and IDU in 12.75% cases. In particular deserves attention IDU transmission path, which is prevalent among men and 89.47% compared with 10.53% in women (p<0.05). Very important is the fact that men more frequently than women are diagnosed with concomitant diseases (viral hepatitis, respiratory, digestive diseases). Thus, one quarter of men with HIV/AIDS (25%) were diagnosed with viral hepatitis versus only 13.08% of women (p<0.05) at both detection and at the initiation of HAART. AIDS was diagnosed in 44.97% of cases on detection and on initiation of HAART AIDS had already almost three quarters of studied patients (73.83%). At HAART initiation, advanced HIV infection (CD4 <200 cells/µL) showed about two-thirds (62.42%) of patients compared with 37.58% at CD4 counts between 350 and 201 cells/µL (p<0.01).

This study showed that more than half (63.09%) of HIV/AIDS-infected patients were detected late, with the number of T-lymphocytes CD4 <350 cells/µL, with or without AIDS related conditions, which determine necessity to improve HIV testing strategies.

